# Neural correlates of fine motor grasping skills: Longitudinal insights into motor cortex activation using fNIRS

**DOI:** 10.1002/brb3.3383

**Published:** 2024-01-11

**Authors:** Xiaoli Li, Minxia Jin, Nan Zhang, Wei Hongman, LianHui Fu, Qi Qi

**Affiliations:** ^1^ Shanghai Yangzhi Rehabilitation Hospital (Shanghai Sunshine Rehabilitation Center) Shanghai China

**Keywords:** cortical activation, functional near‐infrared spectroscopy (fNIRS), longitudinal study, motor learning, motor rehabilitation

## Abstract

**Background:**

Motor learning is essential for performing specific tasks and progresses through distinct stages, including the rapid learning phase (initial skill acquisition), the consolidation phase (skill refinement), and the stable performance phase (skill mastery and maintenance). Understanding the cortical activation dynamics during these stages can guide targeted rehabilitation interventions.

**Methods:**

In this longitudinal randomized controlled trial, functional near‐infrared spectroscopy was used to explore the temporal dynamics of cortical activation in hand‐related motor learning. Thirty‐one healthy right‐handed individuals were randomly assigned to perform either easy or intricate motor tasks with their non‐dominant hand over 10 days. We conducted 10 monitoring sessions to track cortical activation in the right hemisphere (according to lateralization principles, the primary hemisphere for motor control) and evaluated motor proficiency concurrently.

**Results:**

The study delineated three stages of nondominant hand motor learning: rapid learning (days 1 and 2), consolidation (days 3–7), and stable performance (days 8–10). There was a power‐law enhancement of motor skills correlated with learning progression. Sustained activation was observed in the supplementary motor area (SMA) and parietal lobe (PL), whereas activation in the right primary motor cortex (M1R) and dorsolateral prefrontal cortex (PFCR) decreased. These cortical activation patterns exhibited a high correlation with the augmentation of motor proficiency.

**Conclusions:**

The findings suggest that early rehabilitation interventions, such as transcranial magnetic stimulation and transcranial direct current stimulation (tDCS), could be optimally directed at M1 and PFC in the initial stages. In contrast, SMA and PL can be targeted throughout the motor learning process. This research illuminates the path for developing tailored motor rehabilitation interventions based on specific stages of motor learning.

**NEW and NOTEWORTHY:**

In an innovative approach, our study uniquely combines a longitudinal design with the robustness of generalized estimating equations (GEEs). With the synergy of functional near‐infrared spectroscopy (fNIRS) and the Minnesota Manual Dexterity Test (MMDT) paradigm, we precisely trace the evolution of neural resources during complex, real‐world fine‐motor task learning. Centering on right‐handed participants using their nondominant hand magnifies the intricacies of right hemisphere spatial motor processing. We unravel the brain's dynamic response throughout motor learning stages and its potent link to motor skill enhancement. Significantly, our data point toward the early‐phase rehabilitation potential of TMS and transcranial direct current stimulation on the M1 and PFC regions. Concurrently, SMA and PL appear poised to benefit from ongoing interventions during the entire learning curve. Our findings carve a path for refined motor rehabilitation strategies, underscoring the importance of timely noninvasive brain stimulation treatments.

## Graphical abstract

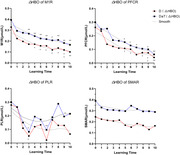

## 1 LIMITATIONS

The primary goal of our investigation was to offer an in‐depth examination of contralateral cortical activation patterns during motor learning using a multitude of tasks. Although our study illuminates vital aspects of this phenomenon, several considerations warrant mention to guide future research endeavors:

Mono‐institutional participant recruitment: Our recruitment strategy was localized to the Tongji University Affiliated Yangzhi Rehabilitation Hospital, which may introduce an element of sample bias, thus possibly restricting the generalizability of our findings. We advocate for future studies to employ broader multi‐institutional recruitment to garner a more heterogeneous sample, thereby extending the insights into neural mechanisms underpinning motor learning.

Depression score discrepancy: Notable differences in depression scores between groups D and displacing and turning (DaT) emerged. Although we excluded this variable from our analytical model, its potential influence on motor learning and neural responses merits further scrutiny in forthcoming investigations.

Data collection cadence: We collected data at strategic junctures—before the inaugural motor learning session and subsequent to each of the ensuing ten sessions. To glean granular insights into the nuances of skill retention and transfer, future studies might consider a more frequent data‐collection paradigm, capturing neural oscillations before and after every session.

Contralateral brain activation focus: Grounded in a vast array of seminal studies (Sosnik et al., 2014), our inquiry predominantly centered on the contralateral brain activation patterns. Although we provided preliminary insights into ipsilateral activation (refer to Appendix), we believe that an exhaustive bilateral brain activation and functional connectivity analysis will significantly augment our understanding of motor learning's neural architecture.

Nondominant hand use: Leveraging the nondominant hand introduced homogeneity among participants. However, executing gestures or sensory stimuli with the nondominant hand might lead to spatial imprecision in responses (Lee et al., 2019), which stands as a potential limitation.

Omission of functional connectivity analysis: Although our focus was pinned on delineating activation trends, we acknowledge that a functional connectivity analysis would have enriched the study's findings, offering an intricate tapestry of the neural networks driving motor learning. This is an avenue we are actively exploring in our subsequent publications.

Exclusion of HbR data: We acknowledge the importance of including HbR data in our study. Although HbR signals might be less sensitive, they offer superior spatial localization. Regrettably, our current research did not encompass this valuable data, which might have enhanced the depth and accuracy of our findings. In future work, we will prioritize incorporating HbR data to provide a more comprehensive representation of neuronal activity.

Anatomical hypotheses consideration: Addressing the anatomical hypotheses necessitated an integrative approach. Yet, we concur that the intricate neural pathways implicated in motor learning might demand a more sophisticated analytical paradigm. Prospective studies should contemplate the integration of advanced neuroimaging techniques or computational models to robustly address this.

## 2 INTRODUCTION

Motor learning, crucial for specific tasks, has a profound impact on daily life, athletic performance, and rehabilitation design. This multifaceted process advances through three distinct stages: the rapid learning phase (initial skill acquisition), the consolidation phase (skill refinement), and the stable performance phase (skill mastery and maintenance) (Dahms et al., 2020; Leech et al., 2022; Marinelli et al., 2017). It is essential to understand these phases, as they provide a framework for deciphering the neural mechanisms underpinning motor learning and its applications in therapeutic contexts.

However, studying motor learning's neural correlates presents inherent challenges due to overlapping processes, which can yield contrasting observations of increasing and decreasing signals (Balsters & Ramnani, 2011; Censor et al., 2012; Shadmehr, 2004). Historically, two phenomena stand out. First, neural recruitment, denoting the engagement of previously inactive neurons, often results in observed increases in brain activity in neuroimaging studies (Karni et al., 1998; Penhune & Doyon, 2002; Shmuelof et al., 2014). On the other hand, reductions in brain activity, especially post‐extensive training, have been documented (Dahms et al., 2020; Ovacik et al., 2022; Sun, 1997; Themanson et al., 2008). The latter is often attributed to the cessation of motor production promotion in specific regions and the potential shifts in synaptic activity within deeper brain structures (Kleim & Jones, 2008). Given these complexities and the dual nature of observed neural responses, there emerges a critical need for innovative therapeutic strategies, paving the way for the application of transcranial magnetic stimulation (TMS) in neural rehabilitation. Given these complexities and the dual nature of observed neural responses, there emerges a critical need for innovative therapeutic strategies, paving the way for the application of TMS in neural rehabilitation.

Our study primarily aims to lay a foundational understanding for TMS treatment in stroke patients, a therapeutic approach that has shown promise in facilitating neural rehabilitation (Chen et al., 2022; Fisicaro et al., 2019). Under normal circumstances, a functional equilibrium is presumed to exist between the cerebral hemispheres, regulated by interhemispheric inhibition (Casula et al., 2021; Sheng et al., 2023). According to the interhemispheric competition model, this balance is disrupted following a stroke or other central nervous system injury diseases; the excitability of the contralesional hemisphere is enhanced, whereas the affected hemisphere experiences abnormally heightened interhemispheric inhibition (Li et al., 2022; Palmer et al., 2019). It is crucial to recognize that the principle underlying TMS stimulation is rooted in the lateralization of motor control, where the modulation of neuronal activity in specific brain regions via TMS seeks to rebalance this altered hemispheric excitability, paving the way for enhanced neural recovery and rehabilitation (Feng et al., 2023; Liepert, 2005; Miniussi & Rossini, 2011; Yuan et al., 2020). These excitability alterations might serve as a significant impediment to functional recovery (de Freitas Zanona et al., 2022; Starosta et al., 2022; Tang et al., 2023). Hence, a plausible strategy for those rehabilitation is to modulate plasticity via repetitive TMS (rTMS), aiming to restore normal activity patterns (Chen et al., 2022; Fisicaro et al., 2019). Although TMS offers promising avenues for direct neural modulation, there is also a pressing need for advanced imaging techniques such as functional near‐infrared spectroscopy (fNIRS) to unravel the intricate neural correlates of motor learning.

Building upon a foundational understanding of TMS, our study seeks to extend its applications and delve deeper into the neural correlates of motor learning. TMS has demonstrated promising potential in facilitating neural rehabilitation, specifically in stroke patients, by establishing a direct connection between the brain and external devices, bypassing peripheral nerves or muscles (Chen et al., 2022; Fisicaro et al., 2019). Clinical outcomes, such as improvements in upper limb Fugl‐Meyer scores, have been observed, substantiating TMS's therapeutic efficacy (Hamaguchi et al., 2020; Ni et al., 2022).

In parallel, our research emphasizes the utility of advanced neuroimaging techniques, with a particular focus on fNIRS. Esteemed for its role in decoding motor behaviors, fNIRS provides a noninvasive, affordable, and highly temporally resolved method that exhibits notable resistance to movement‐induced artifacts, rendering it particularly suitable for studies involving dynamic activities intrinsic to motor learning (Leff et al., 2011; Piper et al., 2014). This method stands out in its ability to address longstanding challenges and discrepancies in our understanding of motor learning, proving its versatility through successful applications across varied contexts, including musical scenarios (Alves Heinze et al., 2019). This noninvasive and highly responsive imaging modality extends its utility beyond general applications, providing critical insights specifically in the realm of motor learning and skill acquisition.

Amidst the evolving landscape of motor learning research, there is an undeniable necessity for meticulous investigations aimed at decrypting the intricate processes and identifying the crucial neural mechanisms underpinning skill acquisition. Various studies (Joshi et al., 2022; Leff et al., 2011; Su et al., 2023) have fortified the vital role of fNIRS in motor behavior research, emphasizing its potential in delineating the dynamics of motor learning. Its utility spans across a myriad of applications, from charting the learning trajectories of piano chords (Alves Heinze et al., 2019) to deciphering the complexities of chopstick use with a nondominant hand (Sawamura et al., 2021) thereby solidifying its credibility.

However, it is imperative to acknowledge that the progressive strides fNIRS has made beyond motor tasks, particularly in addressing cognitive challenges prevalent in specific demographics, such as adolescents grappling with depression. Utilizing fNIRS to monitor hemodynamic responses during the verbal fluency task (VFT) has revealed marked disparities in cortical activation patterns between adolescents with depression and their healthy counterparts (Liu et al., 2022). Recent scholarly pursuits in motor skill learning have consistently shed light on the nuanced cortical activation patterns inherent in skill acquisition. Highlighted in a study (Liu et al., 2022), adolescents with depression exhibited reduced cortical activation in PFC during VFTs. In a parallel vein, the work (Alves Heinze et al., 2019) introduced an ecological perspective, emphasizing the dynamic involvement of the orbitofrontal cortex across various phases of piano playing. Delving deeper into the cerebral mechanisms driving motor skill learning, Hatakenaka et al. (2007) used a pursuit rotor task alongside fNIRS, capturing the transition of cortical activation from the pre‐supplementary motor area (SMA) to the SMA as participants honed their skills. This shift reflects a broader trend observed in motor learning, with early stages marked by pronounced activation in anterior regions such as the PFC and SMA. As proficiency grows and skills consolidate, a posterior shift occurs, with regions like M1 and parietal lobe (PL) gaining prominence. Armed with this knowledge of fNIRS's capabilities in capturing neural dynamics, our study is meticulously designed to investigate the temporal patterns of cortical activation throughout the different stages of motor learning.

However, the field is not without its limitations. Previous research has predominantly adopted a transient, cross‐sectional approach, often sidelining the significance of real‐world tasks and their implications on motor learning (Krakauer et al., 2019; Korivand et al., 2023). Our study seeks to mitigate these shortcomings through a longitudinal exploration, aiming to unravel the temporal dynamics of cortical activation across various stages of motor learning, with a pronounced emphasis on real‐world tasks. Integral to this endeavor is the inclusion of tasks with varying complexity levels, drawing inspiration from the findings (Ovacik et al., 2022) that highlight the profound impact of task complexity on cortical activation patterns. This approach is anticipated to not only illuminate the distinct patterns of neural involvement across different stages of learning but also to offer a comprehensive view on how the brain orchestrates its resources in response to varying challenges. Guided by the hypotheses outlined, we anticipate that this study will not only contribute to the existing body of knowledge on motor learning but also pave the way for advancements in neurorehabilitation, particularly in the realm of noninvasive brain stimulation techniques (Jin et al., 2019). This deliberate and strategic approach to task design and complexity sets the stage for our research hypotheses, aiming to unravel the nuanced relationships among task demands, learning stages, and neural activity.

Motor learning involves regions within the frontal and PLs. Regions like the PFC, M1, SMA, and PL play pivotal roles in orchestrating motor learning processes (Hardwick et al., 2013; Kandel, 1991). The PFC, especially during early sequential learning stages, coordinates with sensory and motor cortices, aligning attention, cognition, and action (Krakauer et al., 2019; Krakauer & Shadmehr, 2006). As learning advances, the role of the SMA in motor planning and sequence timing becomes increasingly pronounced (Kawai et al., 2015).

On the other hand, M1 remains integral for precise hand movement control, with its role in motor execution encoding consistent even during motor adaptation (Schmidt et al., 2018; Wiestler & Diedrichsen, 2013). The PL, a salient sensory region, supports action representation and is crucial in the cortico‐cerebellar circuitry for refining motor skills (Hardie & Wright, 2014).

Our study is designed as a longitudinal exploration, aiming to unravel the temporal dynamics of cortical activation across distinct stages of motor learning with a focus on real‐world tasks. Drawing inspiration from previous studies that underscore the importance of prolonged observational periods (Carey et al., 2005; Park et al., 2022), our approach is tailored to study right‐handed individuals as they perform tasks using their nondominant hand. This specific design choice not only harmonizes with the principles of cerebral lateralization but also aims to enhance the visibility of neural changes during the motor learning process (Liebert et al., 2015; Ruddy & Carson, 2013). Guided by prior studies that have highlighted variable brain region activations during motor learning (Ghilardi et al., 2000), we hypothesize that distinct stages—rapid learning, consolidation, and stable performance—will be characterized by unique cortical activation patterns.

In the domain of motor skill learning research, ensuring a consistent baseline across participants is of paramount importance to accurately infer the effects of the learning process. Our decision to focus on tasks performed with the nondominant hand was rooted in this principle. Utilizing the nondominant hand minimizes the variability introduced by participants’ prior skills and experiences, which can differ substantially when using the dominant hand. By having participants engage in tasks with their nondominant hand, we aimed to establish a more homogenous starting point, where all participants are likely to have limited experience or proficiency. This design choice enhances the internal validity of our study, ensuring that observed neural and behavioral changes can be more confidently attributed to the motor learning process itself, rather than individual differences in prior skill levels. By acknowledging and incorporating these nuances into our study design, we aim to provide a richer, more nuanced understanding of the cortical dynamics underlying motor learning. We believe that this approach not only bolsters the robustness of our findings but also addresses the existing gaps in the literature, offering valuable insights into the intricacies of motor skill acquisition using the nondominant hand.

Factoring in challenges and insights from recent studies, our task‐specific design seeks to enhance the homogeneity of participants’ motor learning baseline and accentuate the observation amplitude of neural changes (Ruddy & Carson, 2013). Drawing from recent research insights, we understand that cortical activation patterns’ evolution is influenced by task complexity and the learning phase. Notably, our study incorporates two tasks of varying difficulty, a design choice guided by prior research (Ovacik et al., 2022). Task complexity plays a significant role in modulating cortical activation patterns. Complex tasks typically demand engagement from a wider array of cortical regions, harnessing more extensive neural networks, whereas simpler tasks might predominantly engage specific areas. In alignment with this understanding, our research design deliberately includes tasks of differential complexity. This not only allows us to discern the nuances in neural involvement contingent on task intricacy but also offers a broader perspective on how the brain orchestrates its resources across varying challenges. We hypothesize that stages such as rapid learning, consolidation, and stable performance will exhibit distinct cortical activation patterns, based on principles of cerebral lateralization and previous studies that have shown variable activation in different brain regions during motor learning (Ghilardi et al., 2000; Liebert et al., 2015).

Based on the theoretical framework and empirical evidence discussed, we posit the following hypotheses to guide our investigation into the neural mechanisms underpinning motor learning. Hypothesis 1 changes in activation patterns hypothesis: Drawing from neuroimaging studies, it is hypothesized that during the early stages of motor learning, there will be a significant increase in activation in PFC and SMA. This is aligned with the finding, where early stages of motor skill acquisition were associated with increased activity in these regions (van Hedel et al., 2021). Conversely, in the later stages of learning, we anticipate a significant increase in activation in M1 and PL, as demonstrated by the posterior shift in cortical activation observed in studies (Krüger et al., 2020; Sun et al., 2022). Task complexity hypothesis: Our hypothesis posits that task complexity will influence changes in brain activation patterns. Specifically, we expect that more complex tasks will engage a broader network of brain regions, whereas simpler tasks will predominantly involve specific brain areas. A study found that the complexity of motor tasks modulates the engagement of different cortical areas, and different patterns of neural activity characterize motor skill performance during acquisition and retention (Beroukhim‐Kay et al., 2022). Through this study, we aim to deepen the understanding of motor learning's neural mechanisms. Such insights could be pivotal for enhancing neurorehabilitation interventions, especially with noninvasive brain stimulation techniques (Jin et al., 2019).

## 3 MATERIALS AND METHODS

The study was a single‐blind, interventional, longitudinal, randomized controlled trial carried out at the Yangzhi Rehabilitation Hospital, which is affiliated with Tongji University in Shanghai, spanning from March 2023 to April 2023. This trial focused on comparing the impact of two distinct motor movement tasks on cortical activation and motor performance of the nondominant upper limb in real‐life settings. The study received approval from the Institutional Review Board at the hospital and complied with the principles of the Helsinki Declaration. All participants involved in the study provided informed consent. The trial was registered at the Chinese Clinical Trial Registry under the registration number ChiCTR2300068624.

### 3.1 Motor learning and assessment task design

**FIGURE 1:**
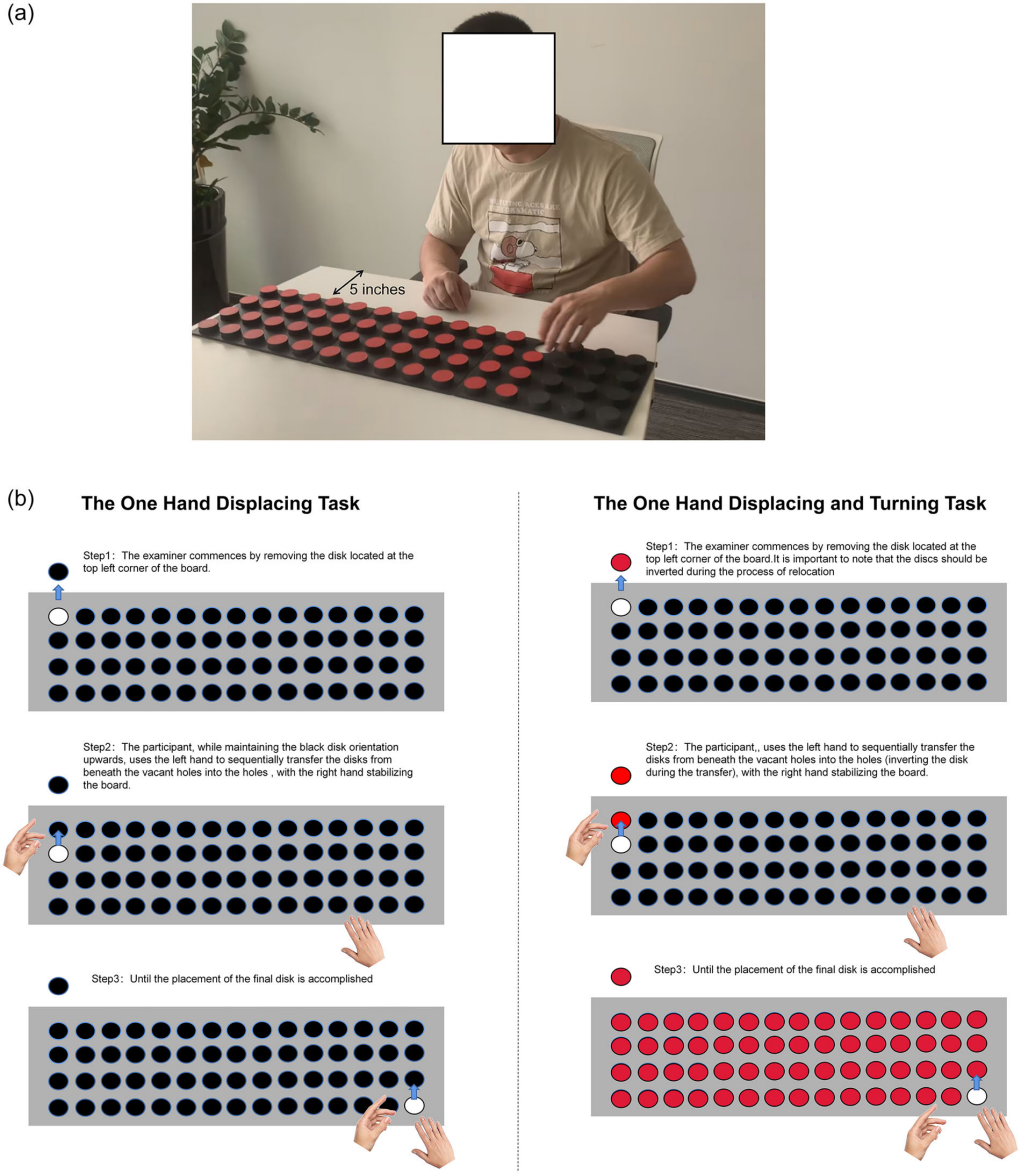
**Minnesota manual dexterity test tasks design diagram**: (a) photographic documentation from the field, (b) schematic representation of the task procedure left: one hand displacing task (D); right: one hand displacing and turning task (DaT).

The study incorporated two tasks from the Minnesota manual dexterity test (MMDT) (Lamers et al., 2013; Ovacik et al., 2022) that represented different levels of complexity—the one hand displacing task (D) and the one hand DaT task. The schematic representation of the participants performing tasks is illustrated in Figure 1a. The MMDT, a well‐recognized clinical hand function assessment tool, imposes time constraints on participants for completing specific fine motor tasks (Lamers et al., 2013; Ovacik et al., 2022). The evaluation outcomes consequently represent the participants’ hand coordination and dexterity levels. This design choice is aligned with the principles of cerebral lateralization and aims to enhance the visibility of neural changes during the motor learning process, thereby providing critical insights for optimizing TMS therapeutic strategies in stroke patients (Kim et al., 2020). Utilizing the nondominant hand minimizes the variability introduced by participants’ prior skills and experiences, which can differ substantially when using the dominant hand (Ghilardi et al., 2000; Liebert et al., 2015).

As the proficiency advances, cortical activation potentially becomes more streamlined, signifying optimized motor control strategies (Bar & DeSouza, 2016; Di Nota & Huhta, 2019). Our endeavor seeks to shed light on how these cortical activation patterns alter as a function of task complexity and learning phase. The task completion time on the MMDT provided an index of motor performance, with a decrease in completion time after training signifying improved hand coordination and dexterity. Thus, a shorter task completion time suggested enhanced motor performance. Our decision to use these two distinct tasks aimed to explore the neurophysiological variations associated with diverse motor skill acquisition and execution. The results would not only enrich our understanding of cortical activation patterns in motor learning but also offer crucial insights for tailoring effective rehabilitation strategies.

The setup begins with positioning a custom‐designed board with numerous holes on a flat table, situated about 5 in. from the edge. Disks that fit these holes are initially placed on the board. The board is then slightly lifted, enabling the disks to drop onto the table, maintaining their alignment in neat rows and columns. If any disks deviate from this grid, they are manually readjusted. The board is then moved forward to rest approximately 1 in. from the edge of the table. This specific placement marks the start of the task.

In the one hand displacing task, participants sat in a predetermined posture and placed cylindrical pegs into holes, following the demonstrated method. The one hand DaT task was a notch higher in complexity, demanding participants to integrate a turning action with the displacing task. These details of tasks are illustrated in Figure 1b.

### 3.2 Participants and randomization

We assessed the eligibility of all interns and staff at the Tongji University Affiliated Yangzhi Rehabilitation Hospital for participation in our study. The inclusion criteria included age between 18 and 35 years, good physical health, normal or corrected‐to‐normal vision, right‐handedness with an Edinburgh Handedness Inventory (EHI) score of >60, and willingness to complete the experiment. We excluded individuals with conditions such as upper limb joint and trunk instability, contracture, fracture, arthritis or other orthopedic diseases, severe cardiovascular, neurological disorders and pulmonary diseases, vertigo, visual or auditory‐vestibular dysfunction, and communicable diseases.

Based on a multivariate analysis of variance approach for repeated measures, we determined an adequate sample size of 30 participants, taking into consideration an anticipated effect size of 0.28 (Lin et al., 2021), a significance level of 0.05 (two‐sided), and a power of 80%. This calculation accounted for an expected dropout rate of 10%; hence, we aimed to recruit a total of 32 participants.

Out of these, we successfully enrolled 32 healthy participants (15 males, 17 females) into our single‐blind parallel‐group randomized controlled trial. Participants were then randomly assigned into two groups using the envelope method, ensuring a balanced distribution.

The two groups were assigned to perform either the one hand displacing task (D) or the one hand DaT task from the Minnesota manual dexterity test (MMDT). These tasks were selected due to their distinct levels of difficulty, thus allowing us to thoroughly investigate the impact of task difficulty on cortical activation during motor learning.

A total of 16 participants were allocated to each task group (D: 8 females, 8 males; DaT: 9 females, 7 males). However, due to personal reasons, one participant from the DaT group could not complete all tasks, leading to a final count of 15 participants in the DaT group and 16 participants in the D group for the data analysis.

Group allocation was conducted using a computer program by an independent statistician. Due to the nature of the intervention, investigators were privy to group assignments, whereas research coordinators and participants were blinded. All participants provided signed informed consent prior to participation. The characteristics of the participants are detailed in Table 1.

**TABLE 1:** Participants demographics and clinical characteristics.

	D	DaT	*p* Value
N	16	15	
**Age (year)**	23.06 ± 3.15	22.13 ± 1.68	.319
**Gender**			.576
*Female*	8 (50%)	9 (60%)	
*Male*	8 (50%)	6 (40%)	
**EHI**	73.23 ± 11.12	75.41 ± 13.11	.621
**BMI,** weight/height **(kg/m^2^)**	21.33 ± 1.84	21.28 ± 1.70	.939
**DASS21**			
*Depression*	4.38 ± 2.58	6.00 ± 2.54	.088
*Stress*	8.12 ± 3.76	6.53 ± 3.25	.218
*Anxiety*	4.62 ± 2.25	4.13 ± 2.50	.615
**(L–R)/R**	−0.04 ± 0.15	0.05 ± 0.20	.142

*Note*: Ages/EHI/BMI are means ± standard deviation; comparisons made by two‐tailed *t*‐test. The EHI (Espírito‐Santo et al., 2017): Handedness patterns are categorized as right‐handed (61–100), ambidextrous (−60 to 60), and left‐handed (−61 to −100). DASS21 (Page et al., 2007): DASS21 is a self‐report instrument that measures depression, anxiety, and stress levels. It has 21 items divided into three dimensions. Respondents select one of four options for each item based on their experiences over the last week. Scores can help assess an individual's levels of depression, anxiety, and stress. In the depression subscale, scores of 10, 14, and 21 represent mild, moderate, and severe depression, respectively. In the anxiety subscale, scores of 8, 10, and 15 represent mild, moderate, and severe anxiety, respectively. In the stress subscale, scores of 15, 19, and 26 represent mild, moderate, and severe stress, respectively. (L–R)/R: This index represents the functional difference between the left and right hand based on the MMDT assessment.

Abbreviations: BMI, body mass index; DaT, displacing and turning; EHI, Edinburgh Handedness Inventory.

### 3.3 Motor learning process

In this study, participants embarked on a 2‐week motor learning cycle, involving a total of 10 motor learning sessions. Each session was conducted daily at a designated time. The training involved executing a specific task 25 times during each session, which was aligned with their assigned motor learning group. The tasks were completed at a natural pace and typically took around 40 min to finish.

In this investigation, our primary endpoint was the shifts in the relative concentration of oxygenated hemoglobin (Δ*HBO*). It should be noted that we opted to employ Δ*HBO* over (Δ*HbR* deoxygenated hemoglobin) given its enhanced sensitivity to task‐induced neuronal activation within our specific experimental paradigm (Strangman et al., 2002). As a secondary measure, we tracked completion times of the Minnesota manual dexterity test (MMDT), which served as a proxy for motor performance of the nondominant upper limb and was thus used as an index of motor performance (Ovacik et al., 2022). These evaluations were systematically conducted throughout the duration of the study to monitor participant progression.

#### 3.3.1 Motor performance assessment

Motor performance was measured using the completion time of MMDT tasks, which served as an indicator of the nondominant upper limb's motor performance (Lamers et al., 2013). Participants were instructed to complete the MMDT task as swiftly as possible. This process was repeated four times, with a 1‐min break between each attempt. The average of these four attempts was then computed to obtain the final motor performance score (Themanson et al., 2008). To clarify the timing of these measurements, data was collected immediately before the first motor learning session and immediately after each of the 10 sessions, resulting in a total of 11 measurements.

#### 3.3.2 Cortical activation assessment

**FIGURE 2:**
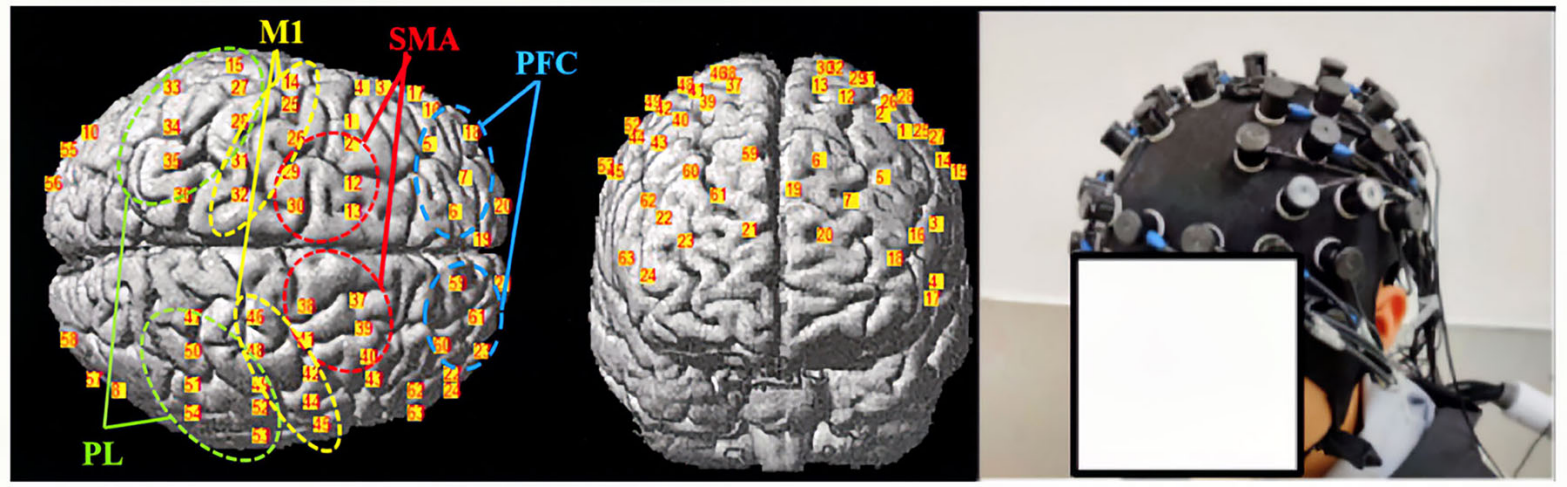
**Head model placement and cortical subdivisions**: from left to right: horizontal channel layout, sagittal channel layout; real‐life participant wearing the device.

We employed a 63‐channel fNIRS system (NirScan, Danyang Huichuang Medical Equipment Co., Ltd., Registration No. 20212061219) with two NIR light wavelengths (730 and 850 nm) and an 11 Hz sampling frequency. Probes were symmetrically arranged on the forehead with a 3 cm spacing between them (Figure 2). Channel locations were obtained using a 3D position‐measuring system (FASTRAC; Polhemus), converted into MNI coordinates (Yu et al., 2020), and projected onto standard brain templates with the NIRS_SPM toolbox (Husain, Yu, et al., 2020; Ye et al., 2009). This spatial registration information facilitated the quantification of activity in various cortical regions (Dahms et al., 2020; Strangman et al., 2002), as specified in the previous paragraph.

**FIGURE 3:**
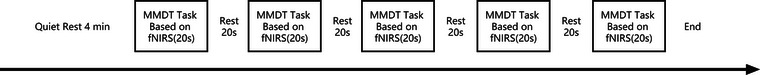
**E‐prime program flowchart**: The direction of the arrow denotes the progression of time throughout the task.

The NirSmart‐6000A multichannel tissue oxygenation monitor (Danyang Huichuang Medical Equipment Co., Ltd.) recorded real‐time cortical activity changes during the task. E‐prime software was employed to maintain fNIRS baseline consistency by fixing acquisition points during data collection. Light intensity brain signals were gathered at a fixed frequency five times, with a total of 11 cortical activation index Δ*HBO* data points collected (1 baseline and 10 after motor learning). The E‐prime‐controlled collection program is detailed in Figure 3.

After data preprocessing, we obtained the average relative concentration changes of oxygenated hemoglobin (Δ*HBO*) as a cortical activation feature index (refer to Section 3.4 for the specific procedure). Given the lateralized pattern of motor control, our study focused on contralateral (right) cortical regions, including SMA, PFC, M1, and PL. The distribution of cortical regions and device placement can be seen in Figure 2 (Strangman et al., 2002). The individual fixed frequency was determined based on initial motor performance.

### 3.4 fNIRS data processing

The preprocessing of fNIRS data, conducted with MATLAB 2014A (The MathWorks Inc.) and the HomER2 processing package, encompassed several stages. This process included (1) the transformation of signal, detection and correction of motion artifacts, and the removal of physiological noise; (2) the application of the Lambert–Beer law for △*HbO* calculations; and (3) the computation of cortical activation task feature values.

Initially, light intensity data was converted into optical density, with a time‐domain baseline correction implemented for the 2 s preceding task onset (Herold et al., 2018). Motion artifact detection and correction were performed to adjust for potential head movements during data acquisition (Husain, Tang, et al., 2020). Following this, the data was standardized to MNI space to ensure precise alignment of cortical regions (Ye et al., 2009, 2020). The spatial registration information allowed the quantification of PFC activity in the left hemisphere (CH 5, 6, 7, and 18) and the right hemisphere (CH 23, 59, 60, and 61), M1 activity in the left hemisphere (CH 14, 25, 26, 31, and 32) and the right hemisphere (CH 42, 44, 45, 46, and 48), SMA activity in the left hemisphere (CH 2, 12, 13, 29, and 30) and the right hemisphere (CH 37, 38, 39, 40, and 41), PL activity in the left hemisphere (CH 15, 27, 28, 33, 34, 35, and 36), and the right hemisphere (CH 47, 49, 50, 51 52, 53, and 54).

A bandpass filter within the frequency range of 0.01–0.1 Hz was used to counteract physiological noise, such as heartbeat and respiration artifacts. The Lambert–Beer law was then employed to convert changes in optical density (Δ*OD*) into cortical Δ*HBO* values, using the absorption spectra of oxygenated hemoglobin and the corresponding scattering constants (Kleim & Jones, 2008). The computed average changes in oxygenated hemoglobin (Δ*HBO*) between the 5th and 22nd seconds post‐task onset across these cortical regions were then used for subsequent analyses (Balsters & Ramnani, 2011; Shadmehr, 2004).

### 3.5 Statistical analysis

Data collection and collation were managed by a dedicated person, and statistical analysis was performed using the R language. Specific packages used in R included “GEEs”’ for generalized estimating equations, and “stats” for *t*‐tests and Wilcoxon signed‐rank tests, among others. All data were continuous variables, expressed in the form of mean ± standard deviation (χ ± SD). We used GEEs to create marginal models, enabling us to statistically analyze the completion time of the MMDT tasks and changes in oxygenated hemoglobin (△*HbO*) at each time point. Importantly, by virtue of GEE's inherent ability to handle repeated measures and correlated observations, the issue of multiple testing has been addressed internally within the model, thus obviating the need for additional multiple comparison corrections. In our analysis, a “significant trend” was identified when the majority of time points, specifically 5 or more out of the total of 10, showed a statistically significant change with a *p*‐value of less than .05.

Independent samples *t*‐test or Wilcoxon signed‐rank test was employed to compare differences in basic characteristics between groups, and the specific test used for each comparison was determined based on the data distribution. The level of statistical significance was set at *p* < .05.

All data were continuous variables, expressed in the form of mean ± standard deviation (χ ± SD). The GEEs were used to establish marginal models for the statistical analysis of MMDT completion time and △*HbO* at each time point. This data analysis method enabled us to map the trajectory of cortical activation changes during motor learning and to explore the potential differences in cortical activation patterns between different task complexities.

In addition to the tests mentioned above, we employed Spearman's rank correlation coefficient to evaluate the strength and direction of association between two continuous variables, namely, the task completion time and the activation of right cortical areas. The absolute value of the Spearman correlation coefficient was interpreted as follows: values between 0.00 and 0.30 indicated a weak correlation, those between 0.30 and 0.60 suggested a moderate correlation, and values from 0.60 to 1.00 represented a strong correlation.

## 4 RESULTS

### 4.1 Motor performance

**FIGURE 4:**
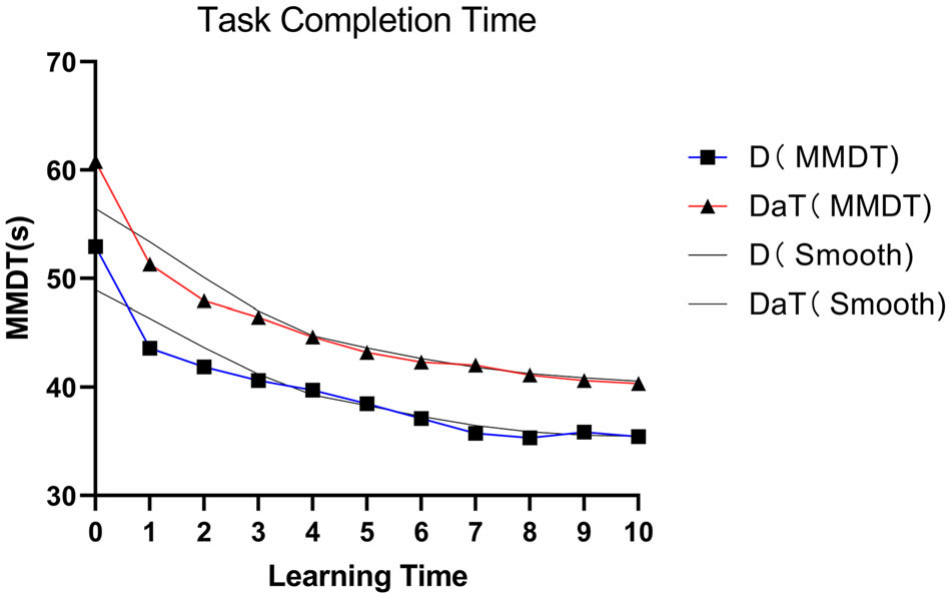
**Trend of task completion time in MMDT** DaT: one hand displacing and turning task group illustrated with blue solid lines; D: one hand displacing task group represented with red solid lines. Vertical axis: mean value of MMDT completion time; horizontal axis: motor learning assessment time points.

The results presented in this section demonstrate that as the number of learning days increased, the completion time for the MMDT task consistently decreased for both the D and DaT groups. A GEE was used to model this relationship, adjusting for age, gender, and EHI factors. The GEE marginal model analysis showed a statistically significant decrease in completion time for both groups from *T*1 to *T*10 (*p* < .01). This indicates that motor learning improved over time for both groups. Further details can be found in Table 2, which displays the differences in MMDT completion times between the D and DaT groups at each assessment point. By using the GEE model to estimate the mean values, a curve was fitted for the completion time of the learning side (left upper limb) task assessment from *T*0 to *T*10. The trend in completion times can be seen in Figure 4.

**TABLE 2:** MMDT time differences between D and displacing and turning (DaT) groups using generalized estimating equation (GEE) (*N* = 31).

*N* = 31	D			DaT		
	*β* (s)	SE	*p* Value	*β* (s)	SE	*p* Value
*T*1 vs. 0	−9.32	.70	<.001	−9.49	1.17	<.001
*T*2 vs. 0	−11.04	1.01	<.001	−12.85	1.16	<.001
*T*3 vs. 0	−12.3067	.91	<.001	−14.36	.89	<.001
*T*4 vs. 0	−13.20	1.18	<.001	−16.16	1.26	<.001
*T*5 vs. 0	−14.44	1.04	<.001	−17.59	1.28	<.001
*T*6 vs. 0	−15.82	1.11	<.001	−18.48	1.25	<.001
*T*7 vs. 0	−17.20	1.38	<.001	−18.77	1.30	<.001
*T*8 vs. 0	−17.60	1.09	<.001	−19.67	1.47	<.001
*T*9 vs. 0	−17.06	.94	<.001	−20.18	1.49	<.001
*T*10 vs. 0	−17.48	1.20	<.001	−20.45	1.41	<.001

*Note*: Based on the GEE model fitting, adjusting for gender, age, and EHI factors. MMDT: Minnesota manual dexterity test task completion time. *β* represents the relationship coefficient. “−”: Indicates a negative relationship.

Abbreviations: EHI, Edinburgh Handedness Inventory; SE, standard error of estimate.

### 4.2 Alterations in brain activation

**FIGURE 5:**
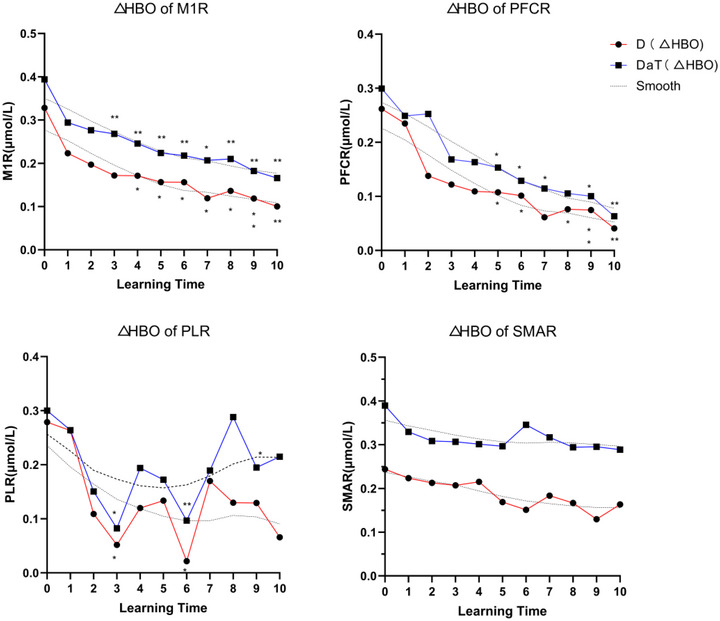
**Activation trends in right hemisphere cortical areas during motor learning** DaT: one hand displacing and turning task group illustrated with blue solid lines; D: one hand displacing task group represented with red solid lines. Vertical axis: The activation of the cerebral cortex was expressed as the change in blood oxygen (Δ*HBO*, μmol/L); horizontal axis: motor learning assessment time points; M1R, right primary motor cortex; PLR: right parietal cortex; PFCR: right dorsolateral prefrontal cortex; SMAR, right supplementary motor area;. “*” indicates *p* < .05, “**” indicates *p* < .01, “***” indicates *p* < .001.

The relationship between the number of days of motor learning and the activation of various right hemisphere cortical areas was analyzed using GEE. The results are shown in Table 3. After adjusting for age, gender, and EHI factors, the GEE marginal model was fitted, revealing different trends in right hemisphere cortical activation with increasing days of motor learning, as illustrated in Figure 5. For right primary motor cortex (M1R), both D and DaT groups showed a decreasing trend from day 5 of motor learning (D: *p* = .011; DaT: *p* = .024), continuing until *T*10 (D at *T*6, *T*7, *T*9, *T*10, *p* < .05, at *T*8 > 0.05; DaT at *T*6, *T*8, *T*9, *T*10, *p* < .05, at *T*7 > 0.05). Right dorsolateral prefrontal cortex (PFCR): D group showed a decreasing trend from day 3 (*p* = .004), and DaT group from day 4 (*p* = .034), with both groups maintaining this trend until *T*10 (D at *T*4, *T*5, *T*6, *T*7, *T*8, *T*9, *T*10, *p* < .05; DaT at *T*5, *T*6, *T*7, *T*8, *T*9, *T*10, *p* < .05). Right supplementary motor area (SMAR): no significant changes in trends (D and DaT at *T*1 to *T*10, *p* > .05). Right parietal cortex (PLR): D group showed a decrease at *T*3, *T*6, *T*10 (*T*3: *p* = .011; *T*6: *p* = .006; *T*10: *p* = .039), DaT group decreased at *T*3, *T*6 (*T*3: *p* = .015; *T*6: *p* = .016), but overall, no significant trends were observed.

**TABLE 3:** Brain activity differences between D and displacing and turning (DaT) groups using generalized estimating equation (GEE) (*N* = 31).

Cortical zone	*TN* vs. *T*0	D			DaT		
		*β* (s)	SE	*p* Value	*β* (s)	SE	*p* Value
M1R	*T*1 vs. 0	−.1002	.1204	.406	−.0999	.0803	.214
	*T*2 vs. 0	−.1307	.0921	.156	−.1175	.0672	.080
	*T*3 vs. 0	−.1576	.0904	.081	−.1261	.118	.285
	*T*4 vs. 0	−.1582	.1035	.127	−.1392	.0839	.097
	*T*5 vs. 0	−.1678	.0659	.011*	−.1703	.0753	.024*
	*T*6 vs. 0	‐.1753	.0756	.020*	−.1665	.0799	.037*
	*T*7 vs. 0	−.2063	.0954	.031*	−.1844	.0957	.054
	*T*8 vs. 0	−.1983	.1125	.078	−.1747	.082	.033*
	*T*9 vs. 0	−.2108	.1043	.043*	−.212	.0729	.004**
	*T*10 vs. 0	−.2203	.0841	.009**	−.2164	.0745	.004**
PFCR	*T*1 vs. 0	−.0269	.0894	.764	−.0506	.0891	.570
	*T*2 vs. 0	−.124	.0859	.149	−.0471	.073	.519
	*T*3 vs. 0	−.14	.0481	.004**	−.1313	.0825	.111
	*T*4 vs. 0	−.1525	.0561	.007**	−.1362	.0641	.034*
	*T*5 vs. 0	−.1542	.0594	.010**	−.1462	.0633	.021*
	*T*6 vs. 0	−.1605	.0588	.006**	−.1706	.0665	.010*
	*T*7 vs. 0	−.2004	.0656	.002**	−.1849	.0513	<.001***
	*T*8 vs. 0	−.1855	.0677	.006**	−.1942	.0678	.004*
	*T*9 vs. 0	−.187	.0638	.003**	−.1992	.0533	<.001***
	*T*10 vs. 0	−.2152	.0786	.006**	−.2357	.0702	.001**
PLR	*T*1 vs. 0	−.0156	.118	.895	−.0361	.118	.760
	*T*2 vs. 0	−.1698	.1419	.231	−.1495	.0791	.059
	*T*3 vs. 0	−.2269	.0893	.011*	−.2176	.0897	.015*
	*T*4 vs. 0	−.1588	.1434	.268	−.1062	.0755	.160
	*T*5 vs. 0	−.1451	.1101	.187	−.1277	.08	.111
	*T*6 vs. 0	−.2571	.0935	.006**	−.2033	.0844	.016*
	*T*7 vs. 0	−.1089	.1232	.377	−.1108	.0806	.170
	*T*8 vs. 0	−.149	.1234	.227	−.0118	.1138	.917
	*T*9 vs. 0	−.1496	.1033	.148	−.1051	.0893	.239
	*T*10 vs. 0	−.2129	.1033	.039*	−.085	.1025	.407
SMAR	*T*1 vs. 0	−.0162	.1337	.903	−.0614	.1389	.658
	*T*2 vs. 0	−.0284	.0963	.768	−.0719	.1033	.486
	*T*3 vs. 0	−.0336	.0812	.679	−.0655	.119	.582
	*T*4 vs. 0	−.0268	.1161	.818	−.0918	.0903	.309
	*T*5 vs. 0	−.0566	.0818	.489	−.0886	.1006	.379
	*T*6 vs. 0	−.0893	.1047	.394	−.0382	.0962	.691
	*T*7 vs. 0	−.0572	.1256	.649	−.0727	.1015	.474
	*T*8 vs. 0	−.0739	.0972	.447	−.0956	.0807	.236
	*T*9 vs. 0	−.1055	.1021	.302	−.0883	.0711	.215
	*T*10 vs. 0	−.0748	.0843	.375	−.1018	.0963	.291

*Note*: Based on the GEE model fitting, adjusting for gender, age, and EHI factors. *β* represents the relationship coefficient. “−”: Indicates a negative relationship. “*”: Indicates *p* < .05, “**”: Indicates *p* < .01, “***”: Indicates *p* < .001.

Abbreviations: M1R, right primary motor cortex; PFCR, right dorsolateral prefrontal cortex; PLR, right parietal cortex; SE, Standard error of estimate; SMAR, right supplementary motor area.

Figure 5 presents the trends in brain activation for the M1R, PFCR, SMAR, PLR over the course of the learning days in both groups. The blue solid lines represent the DaT group, whereas the red solid lines represent the D group. It is evident that as the number of learning days increased, the activation in these specific right hemisphere cortical areas in M1R, PFCR decreased for both groups.

Complementing these findings, Table S1 and Figure S1 chart the brain activity differences in the ipsilateral brain regions of the task‐performing side between D and DaT groups. This Supporting Information section provides additional insights into the bilateral nature of cortical activation during motor learning, underscoring the comprehensive scope of our investigation.

### 4.3 Differences between groups

In Table 4, we present notable findings from applying the GEEs to evaluate the associations between group classifications (DaT and D groups) and activation levels in various cortical regions of the right hemisphere, as well as the MMDT task completion time. This unified approach allowed us to simultaneously assess multiple outcomes, providing robust evidence than analyzing separately. The model was adjusted for age, sex, and EHI factors. The β coefficients represented the mean difference in activation levels (for cortical regions) or completion time (for MMDT task) between the DaT and D groups.

**TABLE 4:** **Difference in right cortex activation between D and** displacing and turning **(DaT) groups (** *N* = **31)**.

ROI	DaT‐D (*β*) (μmol/L)	SE	*p* Value
Time to completion of the MMDT task	5.8208	1.5055	<.001
M1R(ROI)	0.0755	.0315	.016*
PFCR(ROI)	0.0423	.0237	.0746
SMAR(ROI)	0.1303	.0512	.011*
PLR(ROI)	0.0613	.0324	.058

*Note*: ROI: brain regions of interest; DaT‐D (*β*): The difference between the means of the two groups; “*” indicates *p* < .05.

Abbreviations: M1R, right primary motor cortex; PFCR, right dorsolateral prefrontal cortex; PLR, right parietal cortex; SE, Standard error of estimate; SMAR, right supplementary motor area.

Task difficulty did not significantly impact the activation levels in the PFCR (*β* = .0423, SE = .0237, *p* = .0746) and PLR (*β* = .0613, SE = .0324, *p* = .058). Contrarily, significant group differences were observed in the activation levels in the SMAR (*β* = .1303, SE = .0512, *p* = .011) and M1R (*β* = .0755, SE = .0315, *p* = .016). Additionally, the group classification showed a significant association with the MMDT task completion time (*β* = 5.8208, SE = 1.5055, *p* < .001), indicating that the DaT group took a longer time to complete the MMDT task compared to the D group.

### 4.4 Association between brain activation and motor performance

**FIGURE 6:**
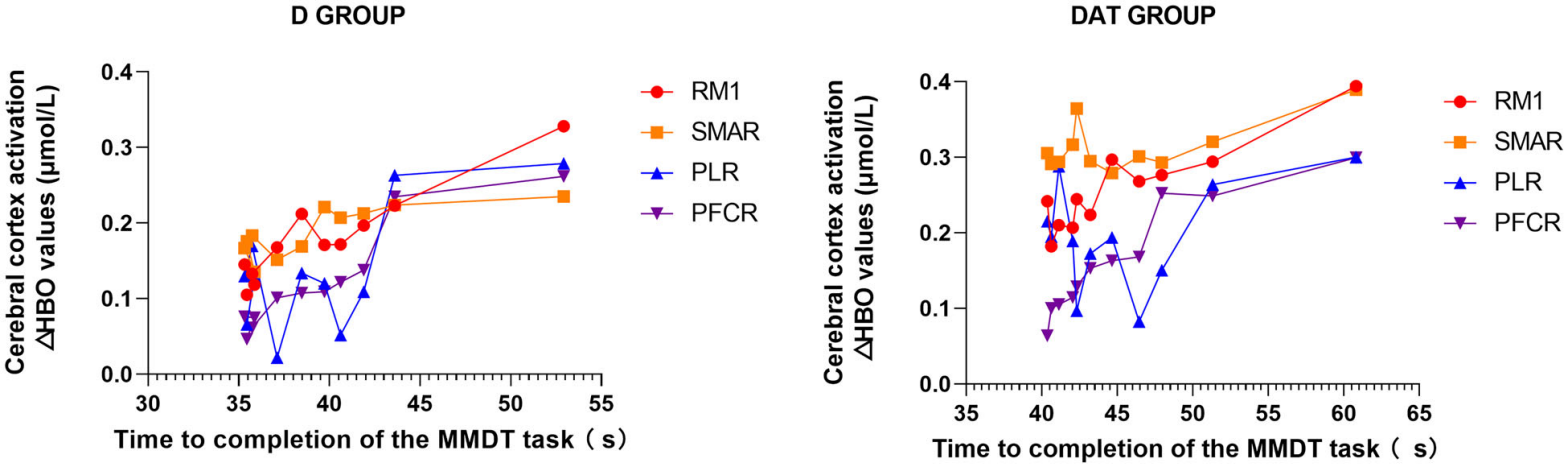
Temporal fluctuations in blood oxygenation levels during cortical activation and their association with motor performance. The *x*‐axis represents the completion time (s) of the MMDT task for the left upper limb (learning side), and the *y*‐axis indicates the activation (μmol/L) of the right cortical areas during the motor learning assessment of the learning side (left upper limb). M1R, right primary motor cortex; PFCR, right dorsolateral prefrontal cortex; PLR, right parietal cortex; SMAR, right supplementary motor area.

Figure 6 illustrates the temporal fluctuations in blood oxygenation levels during cortical activation and their association with motor performance. For the D group, the correlation coefficients were as follows: M1R = 0.88 (*p* < .05), SMAR = 0.77 (*p* < .05), PLR = 0.26, and PFCR = 0.95 (*p* < .05). For the DaT group, the correlation coefficients were as follows: M1R = 0.83 (*p* < .05), SMAR = 0.29 (*p* > .05), PLR = −0.02 (*p* > .05), and PFCR = 0.99 (*p* < .05).

On comparing the correlation coefficients between the D and DaT groups, some key differences were observed. The activation of M1R and PFCR showed a strong correlation with motor performance in both groups, although the correlation coefficients were slightly higher in the D group for M1R and in the DaT group for PFCR.

Conversely, the correlation between SMAR activation and motor performance varied substantially between the D and DaT groups. In the D group, SMAR activation was strongly correlated with motor performance, whereas in the DaT group, it showed a weak correlation.

In terms of PLR, its activation was weakly correlated with motor performance in the D group, whereas in the DaT group, there was no significant correlation.

These differences between the groups highlight the variability in cortical activation patterns relative to motor performance, underscoring the importance of individualized assessments and targeted interventions in motor learning tasks.

## 5 DISCUSSION

The aim of this study was to delve into the intricate temporal dynamics of cortical activation across the spectrum of motor learning stages, specifically when individuals engage in real‐world tasks. A nuanced understanding of how neural mechanisms control lateralized functions during motor learning can have profound implications, especially in the context of optimizing neurorehabilitation interventions. Such understanding can pave the way for more effective utilization of noninvasive brain stimulation techniques, including TMS and transcranial direct current stimulation (tDCS).

This perspective is consistent with the findings (Jin et al., 2019), which assert that leveraging TMS for the treatment of conditions like stroke predominantly relies on the principles of interhemispheric inhibition and lateralized control. This intricate relationship between neuromotor disorders, such as stroke, and the therapeutic application of TMS is further elucidated by the same study. Specifically, the results from Jin et al. highlight the promising potential of rTMS combined with motor training as a means to bolster motor functionalities. Their investigations unveiled significant modifications in interhemispheric lateralization, particularly emphasizing shifts in power spectral density and the efficiency of information transmission between the central cortices of both brain hemispheres.

By actively employing these principles, rehabilitation strategies can be tailored to target and modulate neural activity within specific brain regions. This targeted approach can aid in restoring balanced neural function and consequently, enhancing motor control capabilities. Another salient point to consider is the pivotal role of fNIRS in evaluating complex bimanual motor skills. For instance, its application in the domain of surgical procedures underscores the versatility and significance of fNIRS in a variety of motor tasks, as highlighted by the study (Nemani et al., 2018).

In our study, we had the privilege of working with 31 right‐handed participants. These individuals were categorized into two separate groups, primarily based on the intricacy of the motor learning tasks assigned to them. Group D partook in the “One Hand Displacing” task, which can be described as a relatively straightforward motor activity. In contrast, Group DaT was tasked with the “One Hand Displacing and Turning” task. This latter task inherently possessed an added degree of complexity, attributed to the turning action it mandated. It is worth noting that participants in both groups were subjected to 10 sessions of rigorous motor learning training. The primary focus of this training regimen was to hone the coordination and dexterity of their nondominant (left) upper limb.

### 5.1 The effect of action learning duration on motor performance trends

As motor learning progressed from the 5th to the 10th session, both the D and DaT groups displayed a significant decrease in motor performance (*p* < .001). For the D group, a plateau phase was achieved on the seventh day, whereas for the DaT group, this phase was reached on the ninth day. Here, we define the “stable period” as the phase in which no significant improvements in motor performance were observed.

This observed trend in motor performance aligns with the well‐established theory of motor learning stages. The process of left upper limb (learning side) motor learning can be divided into three stages: the rapid learning period (sessions 1 and 2), the consolidation learning period (sessions 3–7), and the stable plateau period (sessions 8–10) (Doyon et al., 2009).

Contrasting with Newell's proposal that skill improvement would slow down with each doubling of practice repetitions (Newell & Rosenbloom, 1980), our study observed a similar pattern within ten sessions conducted over 2 weeks. Each session was at a freely chosen speed for left nondominant hand action learning training. We speculate that the rapid improvement in motor performance during the rapid learning period might be due to the establishment of central‐related synaptic connections at this stage. During the consolidation period, repeated practice leads to a decrease in the synaptic establishment rate, resulting in slower progress in motor performance. In the stable plateau period, synaptic connections have likely reached a relatively stable state, and motor performance shows minimal changes (Cohen et al., 1990).

### 5.2 The effect of action learning duration on the changing trend of right brain activation

#### 5.2.1 Trends in activation changes of the right primary motor cortex (M1R)

Both the simple group (D) and the complex group (DaT) showed a decline in M1R activation from the fifth day of action learning (D: *p* = .011; DaT: *p* = .024). The decline stopped for the D group on the eighth day (*p* > .05) and for the DaT group on the seventh day (*p* > .05). Subsequently, D and DaT showed a continuous decline from *T*9 and *T*8, respectively, to *T*10 (D: *T*6, *T*7, *T*9, *T*10, *p* < .05; DaT: *T*6, *T*8, *T*9, *T*10, *p* < .05). M1R activation shows a near‐power‐function decline trend with increasing learning days. The primary motor area (M1) can project directly to the spinal motor neurons and is closely related to action execution control (Penhune & Steele, 2012). M1 has the function of muscle synergy; as learning progresses, action skills become more proficient, and the demand for synergistic muscles decreases, reducing involvement (Kawai et al., 2015). This indicates that M1 is responsible for action execution in action learning, consistent with previous experimental studies (Penhune & Steele, 2012). This may be because repeated training induces a transient increase in dendrites in the M1 area neurons; after skills become more proficient, the adaptive increase in M1 neurons reaches saturation, and synaptic excitability gradually decreases (Hamasaki et al., 2019; Miller, 2000; Withers & Greenough, 1989). That is, after truly mastering action skills, the involvement of M1 decreases (Kawai et al., 2015).

In order to further elucidate the underlying mechanisms of the observed decline in M1R activation, future studies might consider the functional connectivity of M1R with other brain regions such as the prefrontal and cerebellar areas. This could potentially illuminate the cooperative functioning between brain regions during the process of motor learning and the specific role of M1R within this context.

#### 5.2.2 Trends in activation changes of the right dorsolateral prefrontal cortex (PFCR)

In the PFCR region, the simple group (D) exhibited a decreasing trend from day 3 (*p* = .004), whereas the complex group (DaT) showed a decreasing trend from day 4 (*p* = .034). Both groups’ decline continued until *T*10 (D: *T*4, *T*5, *T*6, *T*7, *T*8, *T*9, *T*10, *p* < .05; DaT: *T*5, *T*6, *T*7, *T*8, *T*9, *T*10, and *p* < .05). The activation of PFCR displayed a power‐law decline with the increase in learning days. The PFC is involved in multiple neural networks and possesses functions such as task acquisition, plasticity, automation, neural activity, flexibility, and long‐term storage (Miller, 2000). In line with our observations, studies in surgical motor skills have shown that the PFC, along with other regions like the primary motor cortex and SMA, plays a crucial role in the acquisition and refinement of complex motor tasks, further highlighting the overarching significance of these regions in motor learning (Nemani et al., 2018). Studies have shown that during the early stages of motor learning, increased PFC activation reflects the cognitive demands of working memory and explicit knowledge storage for newly learned tasks. As skill improves, PFC activity gradually decreases because, after reaching automation, task execution no longer requires additional task information acquisition and cognitive control (Elston et al., 2011; Friedman & Robbins, 2022; O'Reilly & Frank, 2006; Polskaia et al., 2023; Rodriguez et al., 2018). This implies that the PFC is responsible for higher cognitive functions in motor learning, mainly performing action recognition and representing motivation in action control (Xu et al., 2022). In line with this, research in a musical context has shown dynamic changes in the prefrontal hemodynamics as individuals learn a piano chord progression, highlighting the central role the PFC plays not only in fine motor grasping skills but also in more complex motor tasks such as playing an instrument (Alves Heinze et al., 2019). Consistent with these findings, recent advancements have showcased the adaptability of fNIRS in discerning cortical activations in intricate bimanual tasks such as laparoscopic surgeries. Here, the PFC and associated regions have been identified to play a pivotal role in the modulation and control of these intricate motor tasks, reaffirming the significance of the PFC in diverse motor functions (Nemani et al., 2018). This notion of adaptability and neural plasticity in motor learning is further supported by studies that investigated training interventions for the nondominant hand. For instance, a recent study on chopstick operation training demonstrated significant improvements in nondominant hand skills after a 6‐week training regimen, with fNIRS findings illustrating the associated cortical adaptations (Sawamura et al., 2021). Our findings resonate with studies that investigated cognitive deficits in different populations. For instance, in adolescents with depression, abnormal activation patterns were observed, with significantly less cortical activation in the hemodynamic responses of △*HBO* primarily in the PFC during tasks (Liu et al., 2022). Such parallels emphasize the robustness and versatility of fNIRS in capturing nuanced neural activations across varied tasks and populations. This mechanism may originate from the PFC's unique neuroanatomical connection distribution, facilitating participation in multilevel neural networks (Friedman & Robbins, 2022), and playing a role in maintaining working memory targets and the flexibility of the multiple demand network (O'Reilly & Frank, 2006).

It is worth noting that our results may suggest a holistic shift in the brain during the process of motor learning. The observed decline in PFCR activation might represent a shift from reliance on explicit knowledge storage and cognitive control in the prefrontal cortex toward a more implicit memory and automatic action control mediated by other regions such as the cerebellum and basal ganglia. Further studies are needed to validate this hypothesis.

#### 5.2.3 Trends in activation changes of the right parietal lobe cortex (PLR)

In the PLR region, a decreasing trend was observed in the simple group (D) at *T*3, *T*6, and *T*10 (*T*3: *p* = .011; *T*6: *p* = .006; *T*10: *p* = .039), whereas the complex group (DaT) showed a decreasing trend at *T*3 and *T*6 (*T*3: *p* = .015; *T*6: *p* = .016). Activation of the right parietal lobe cortex (PLR) demonstrated a zigzag pattern of continuous activation as the number of learning days increased. According to the sensorimotor learning model proposed by Doyon et al., the PL cortex is a critical component of the cortico‐cerebellar network, emphasizing the role of sensory feedback in motor learning and being closely related to the predictive and corrective mechanisms involved in motor learning adaptation (Hänggi et al., 2015). Studies have shown that in the early stages of motor learning, the PL works closely with the cerebellum to correct learned actions through sensory feedback, thus resulting in an increase in early activation levels. As the skill is mastered, the brain's sensory feedback demand decreases, and the activation level decreases. However, as the predictive and corrective mechanisms associated with motor adaptation are engaged and motor performance further improves, parietal activation levels increase again (Doyon et al., 2003; Hänggi et al., 2015). This suggests that the PL is responsible for correcting and adjusting task accuracy during motor learning (Krakauer & Mazzoni, 2011). The specific mechanism is based on the framework of sensorimotor task performance improvement in the cortico‐cerebellar network model. The PL is an essential sensory input region that modulates the overall excitability of neurons, making the neuronal activity gradually match the cortical activation required for the corresponding sensory input level. As this demand decreases, parietal activation decreases. With the improvement of motor performance, the predictive and corrective mechanisms involving the PL are initiated, and new stimulus‐response representations emerge within the parietal cortex. This leads to a change in the encoding mode of cortical motor learning and an increase in parietal activation (Grol et al., 2006, 2009; Orban et al., 2010).

Understanding the role and activation patterns of PLR in motor learning might have significant implications for neurorehabilitation. For instance, specific neuro‐modulation techniques targeting PLR could potentially enhance the recovery outcomes for patients with motor learning deficits. Further research in this direction is warranted.

#### 5.2.4 Trends in activation changes of the right supplementary motor area (SMAR)

In both groups of subjects, no significant upward or downward trend in activation was observed in the SMAR region (*p* > .05). Throughout the learning process, the SMAR exhibited a continuous activation state. The function of the SMA is relatively complex, interconnected with multiple central nervous structures, including the basal ganglia, cerebellum, limbic system, thalamus, superior parietal lobule, frontal lobe, and spinal branches; moreover, the SMA is also an essential component of the cortico‐basal ganglia–cerebellar circuit (Mushiake et al., 1991; Rahimpour et al., 2022). According to related research, the SMA is closely connected with the deep nuclei of the basal ganglia, and the transmission of information between the basal ganglia and the extrapyramidal system influences each other, playing a crucial role in adjusting body posture and coordinating movement (Akkal et al., 2007). In motor learning, the mechanism involving the SMAR may be complex, and no definitive pattern was observed in this study.

### 5.3 Differences between groups and comparative analysis

Our study unveils distinct cortical activation patterns between the simple task group (D) and the complex task group (DaT), which corroborate the significant impact of task complexity on motor learning dynamics. Participants in the DaT group, confronted with more intricate tasks, exhibited a noticeably protracted decline in M1R and PFCR activations when contrasted with their counterparts in the D group (Di Nota & Huhta, 2019). Such findings suggest that the DaT group experienced enhanced cognitive demands inherent to complex motor tasks, resulting in the extended involvement of relevant brain regions.

The DaT group's tasks, with their inherent complexity, undoubtedly necessitated an amplified integration of sensory feedback and cognitive processing. This conclusion is further substantiated by the heightened engagement of the parietal and prefrontal regions observed in this group. Conversely, the D group's tasks, in their simplicity, seemingly steered participants toward a swifter progression toward motor response automatization, culminating in a more pronounced activation of the primary motor area.

The juxtaposition of neural engagement across both groups unravels the fact that the complexity of motor tasks not only differentiates which brain regions are predominantly engaged but also delineates the depth and duration of such engagements. Specifically, the DaT group, grappling with compounded task intricacies, exhibited a pronounced adaptability in regions pivotal for cognitive processing and feedback integration. This contrasts sharply with the D group, where rapid skill consolidation seemed to overshadow extended neural adaptability.

Our findings, in essence, underscore the pivotal role of task complexity in steering the trajectory of motor learning. This insight is paramount when fashioning motor learning paradigms, both in avant‐garde research environments and pragmatic clinical settings. Given these differential neural activations based on task complexities, future research, echoing the sentiments of the findings (Li et al., 2020), should delve into the intricate interregional functional connectivities discerned in varied task types. Such endeavors can illuminate the neural dynamics and interplays at the helm of motor tasks varying in complexity, thereby offering a richer understanding of motor learning.

Following this comparative discourse on the D and DaT groups, our discourse segues into the intricate relationship between brain activation shifts and their tangible manifestations in motor performance enhancements.

### 5.4 Association between brain activation and motor performance

Figure 6 provides a compelling depiction of the nuanced association between cortical activation in the right hemisphere and motor performance of the left upper limb. The variations in blood oxygenation levels during cortical activation offer a lens into the brain's adaptive processes during motor skill acquisition.

The near‐power‐function decline trend in M1R and PFCR activation for the D group aligns with the learning curve frequently observed in motor skill development. Such a pattern is indicative of the brain navigating the cognitive complexities during the initial phases, gradually transitioning to a phase where the motor skill becomes more automated, thus underscoring the dynamic nature of neural adaptations during learning (Dayan & Cohen, 2011).

Contrastingly, for the DaT group, the robustness of the M1R and PFCR correlation with motor performance brings forth nuances that are in resonance with Di Nota and Huhta (2019). The slightly heightened activation, particularly in PFCR for the DaT group, might hint at an extended engagement of the dorsolateral prefrontal cortex, implying a more prolonged cognitive orchestration and aligning with the findings from Hardwick et al. (2013).

SMAR's divergent patterns between the two groups suggest its intricate role in motor learning. In the D group, SMAR's strong correlation with motor performance can be seen as a testament to its role in information integration from various brain areas, optimizing motor performance. However, its weak correlation in the DaT group indicates a broader neural network engagement, potentially due to the multifaceted demands of the DaT tasks.

The oscillatory pattern of PLR activation draws attention to its versatile function. The zigzag pattern observed could be an outcome of an intricate dance among prediction, adjustment, and correction mechanisms, reaffirming the significance of the parietal cortex in motor skill refinement (Desmurget et al., 1999).

## 6 CONCLUSION

Drawing from the foundational principles of TMS‐induced lateralization, our study endeavored to systematically evaluate the implications of the duration of learning on cortical neural activation and concurrent alterations in motor performance during nondominant (left) upper limb motor learning among right‐handed participants. A cohort of 31 right‐handed individuals was stratified into distinct groups and underwent an intensive 10‐day regimen of nondominant upper limb motor learning exercises. Observational data elucidated a progressive enhancement in upper limb motor proficiency concomitant with a steady decrement in activation intensities within the M1R and the PFCR.

The trajectory of this motor performance enhancement resonates robustly with the stratified stages of movement learning theory. The left upper limb (learning side) motor learning trajectory was categorized into an accelerated learning phase (spanning days 1 and 2), followed by a consolidation phase (spanning days 3–7), culminating in a stabilization plateau (days 8–10). These empirical observations harmoniously align with Newell's seminal 1980 doctrine, suggesting that motor learning performance evolution adheres to the power law of practice. Notably, analogs curves were discerned through a methodological framework encompassing 10 training iterations over a fortnight, each demanding nondominant hand movement learning exercises executed at a participant‐determined pace.

Furthermore, the present research delineates activation trends within four pivotal regions of the right cerebral hemisphere amid the motor learning continuum. These empirical insights significantly augment contemporary comprehension of the neural architectures and pathways implicated in nondominant upper limb motor learning. Thus, this research enriches the prevailing narrative on neuroplasticity and the neural substrates underpinning motor skill acquisition and mastery, thereby informing the conceptualization of optimized rehabilitation modalities for individuals grappling with motor dysfunctions.

In summation, the present investigation offers preliminary insights into the intricate interplay between neural activation dynamics and motor performance during nondominant upper limb motor learning, scaffolded by the principles of TMS‐induced lateralization. This repository of knowledge burgeons the potential for the evolution of bespoke rehabilitation blueprints tailored for right‐handed individuals. Nevertheless, the groundbreaking nature of our findings underscores the imperative for subsequent research endeavors to further crystallize and corroborate these nascent insights. In conclusion, our study has provided initial insights into the changes in neural activation and motor performance during nondominant upper limb motor learning, and underpinned by the principles of TMS‐induced lateralization, unveils the intricate relationship between neural activation and motor performance during nondominant upper limb motor learning. This knowledge holds significant promise for crafting personalized rehabilitation strategies for right‐handed individuals. Although our study introduces innovative methods closely tied to clinical practice and provides initial evidence, it highlights the necessity for further research to expand upon and substantiate these preliminary insights.

## AUTHOR CONTRIBUTIONS

**Xiaoli Li**: Conceptualization; investigation; methodology; validation; software; formal analysis; data curation; supervision; project administration; visualization; writing—original draft; writing—review and editing; resources. **Minxia Jin**: Writing—review and editing; methodology; resources. **Nan Zhang**: Writing—review and editing; resources. **LianHui Fu**: Writing—review and editing; resources. **Hongman Wei**: Methodology; resources. **Qi Qi**: Funding acquisition; project administration; resources; formal analysis; conceptualization; writing—review and editing; methodology; investigation.

## CONFLICT OF INTEREST STATEMENT

The authors declare no conflicts of interest.

## FUNDING INFORMATION

No funding or sponsorship was received for this study or publication of this article.

### PEER REVIEW

The peer review history for this article is available at https://publons.com/publon/10.1002/brb3.3383.

## Supporting information

Figure S1 Brain activation on the task‐performing side ipsilateral brain regions.
DaT: one hand displacing and turning task group illustrated with blue solid lines; D: one hand displacing task group represented with red solid lines. Vertical axis: The activation of the cerebral cortex was expressed as the change in blood oxygen (Δ*HBO*, μmol/L); horizontal axis: motor learning assessment time points. M1L: left primary motor cortex; SMAL: left supplementary motor area; PLL: left parietal cortex; PFCL: left dorsolateral prefrontal cortex. “*” indicates *p* < .05, “**” indicates *p* < .01, “***” indicates *p* < .001.

Table S1 Brain activity differences of the task‐performing side ipsilateral brain regions between D and DaT groups using GEE (*N* = 31) based on the GEE model fitting, adjusting for gender, age, and EHI factors. M1L: left primary motor cortex; SMAL: left supplementary motor area; PLL: left parietal cortex; PFCL: left dorsolateral prefrontal cortex.** β represents the relationship coefficient. “−”: Indicates a negative relationship. “*”: Indicates *p* < .05, “**”: Indicates *p* < .01, “***”: Indicates *p* < .001. SE: Standard error of estimate.
The supplementary materials clearly chart the activation trajectories in ipsilateral cortical regions (M1L, PFCL, SMAL, and PLL) across learning days in both experimental groups. These trajectories are visually represented by solid lines: blue for the DaT group and red for the D group. Notably, we observed a noticeable reduction in activation levels in the M1L and PFCL regions of the right hemisphere, consistent across both groups.
We intentionally focused our main text on contralateral cortical activation patterns, vital for understanding motor learning in the context of potential transcranial magnetic stimulation (TMS) and transcranial direct current stimulation (tDCS) interventions. This approach aligns with motor control lateralization principles, aiming to enhance rehabilitative strategies. However, recognizing the crucial role of ipsilateral regions in motor learning, we have included supplementary data to offer a comprehensive view, ensuring that our research is transparent and complete.
We are ready to further explore this dataset, with a forthcoming publication dedicated to examining bilateral cortical interactions and functional connectivity within the motor learning context. This separate research effort aims to strengthen our knowledge, offering detailed insights into the intricate relationships between cortical regions during motor learning, and informing neuro‐rehabilitative interventions. Our dedication to progressing this field is steadfast, and we believe that this phased approach in sharing our findings will maintain scientific integrity, foster targeted rehabilitation methods, and ultimately benefit both the scientific community and patient care.

## Data Availability

Raw and processed data supporting the findings of this study are available from the corresponding author, Qi Qi, upon reasonable request.
