# Supplementary figures and images for: Neural correlates of fine motor grasping skills: Longitudinal insights into motor cortex activation using fNIRS
Source: Brain Behav. 2024 Jan 11;14(1):e3383. doi: 10.1002/brb3.3383 (PMC10784192; doi:10.1002/brb3.3383)

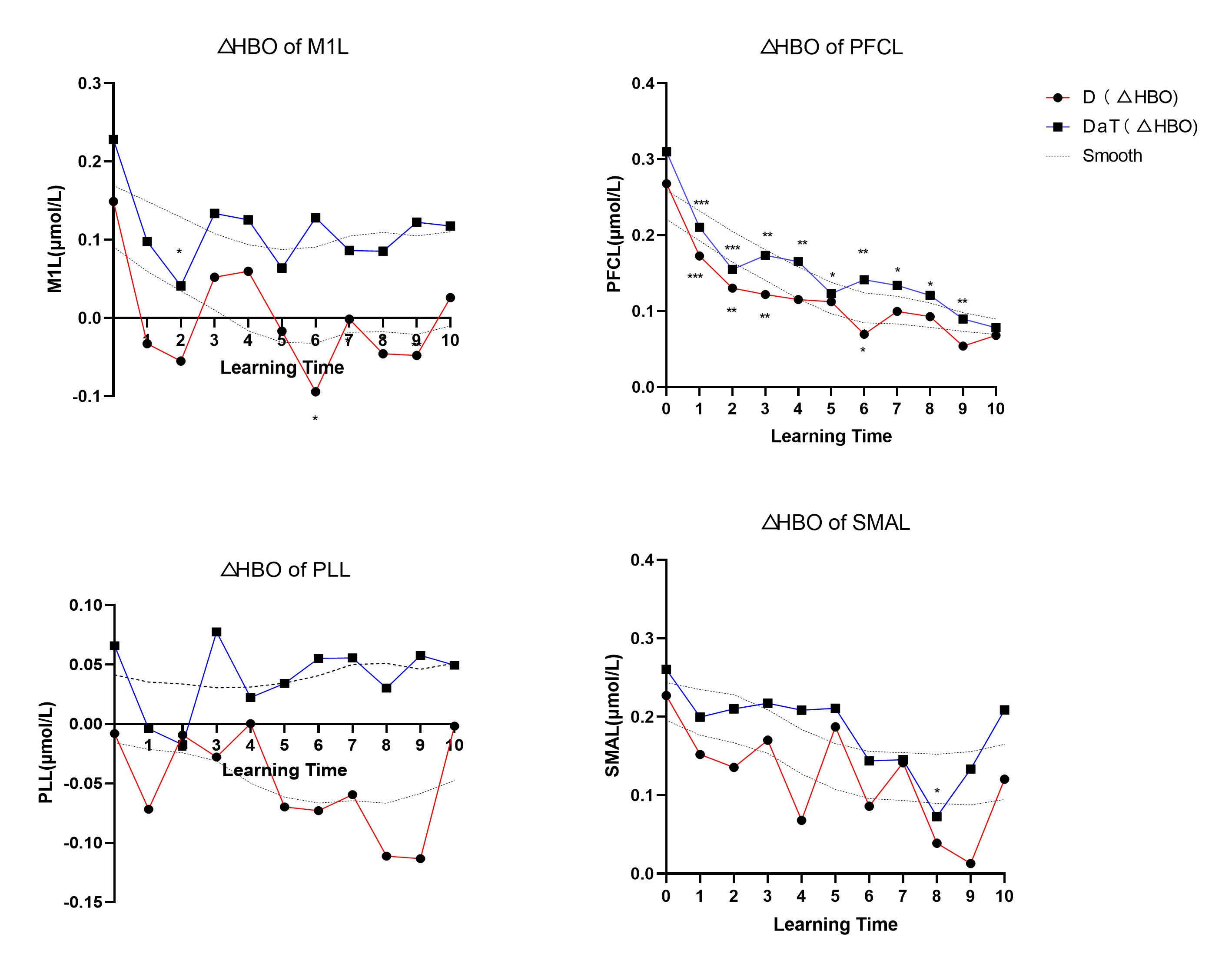

Supplement: Supplementary file 1 — Figure S1 Brain activation on the task‐performing side ipsilateral brain regions. DaT: one hand displacing and turning task group illustrated with blue solid lines; D: one hand displacing task group represented with red solid lines. Vertical axis: The activation of the cerebral cortex was expressed as the change in blood oxygen (ΔHBO, μmol/L); horizontal axis: motor learning assessment time points. M1L: left primary motor cortex; SMAL: left supplementary motor area; PLL: left parietal cortex; PFCL: left dorsolateral prefrontal cortex. “*” indicates p < .05, “**” indicates p < .01, “***” indicates p < .001. [file BRB3-14-e3383-s002.tif]
