# Supplementary material for: Neural correlates of fine motor grasping skills: Longitudinal insights into motor cortex activation using fNIRS
Source: Brain Behav. 2024 Jan 11;14(1):e3383. doi: 10.1002/brb3.3383 (PMC10784192; doi:10.1002/brb3.3383)
Supplement: Supplementary file 2 — Table S1 Brain activity differences of the task‐performing side ipsilateral brain regions between D and DaT groups using GEE (N = 31) based on the GEE model fitting, adjusting for gender, age, and EHI factors. M1L: left primary motor cortex; SMAL: left supplementary motor area; PLL: left parietal cortex; PFCL: left dorsolateral prefrontal cortex.** β represents the relationship coefficient. “−”: Indicates a negative relationship. “*”: Indicates p < .05, “**”: Indicates p < .01, “***”: Indicates p < .001. SE: Standard error of estimate. The supplementary materials clearly chart the activation trajectories in ipsilateral cortical regions (M1L, PFCL, SMAL, and PLL) across learning days in both experimental groups. These trajectories are visually represented by solid lines: blue for the DaT group and red for the D group. Notably, we observed a noticeable reduction in activation levels in the M1L and PFCL regions of the right hemisphere, consistent across both groups. We intentionally focused our main text on contralateral cortical activation patterns, vital for understanding motor learning in the context of potential transcranial magnetic stimulation (TMS) and transcranial direct current stimulation (tDCS) interventions. This approach aligns with motor control lateralization principles, aiming to enhance rehabilitative strategies. However, recognizing the crucial role of ipsilateral regions in motor learning, we have included supplementary data to offer a comprehensive view, ensuring that our research is transparent and complete. We are ready to further explore this dataset, with a forthcoming publication dedicated to examining bilateral cortical interactions and functional connectivity within the motor learning context. This separate research effort aims to strengthen our knowledge, offering detailed insights into the intricate relationships between cortical regions during motor learning, and informing neuro‐rehabilitative interventions. Our dedication to progressing t [file BRB3-14-e3383-s001.docx]

# APPENDIX


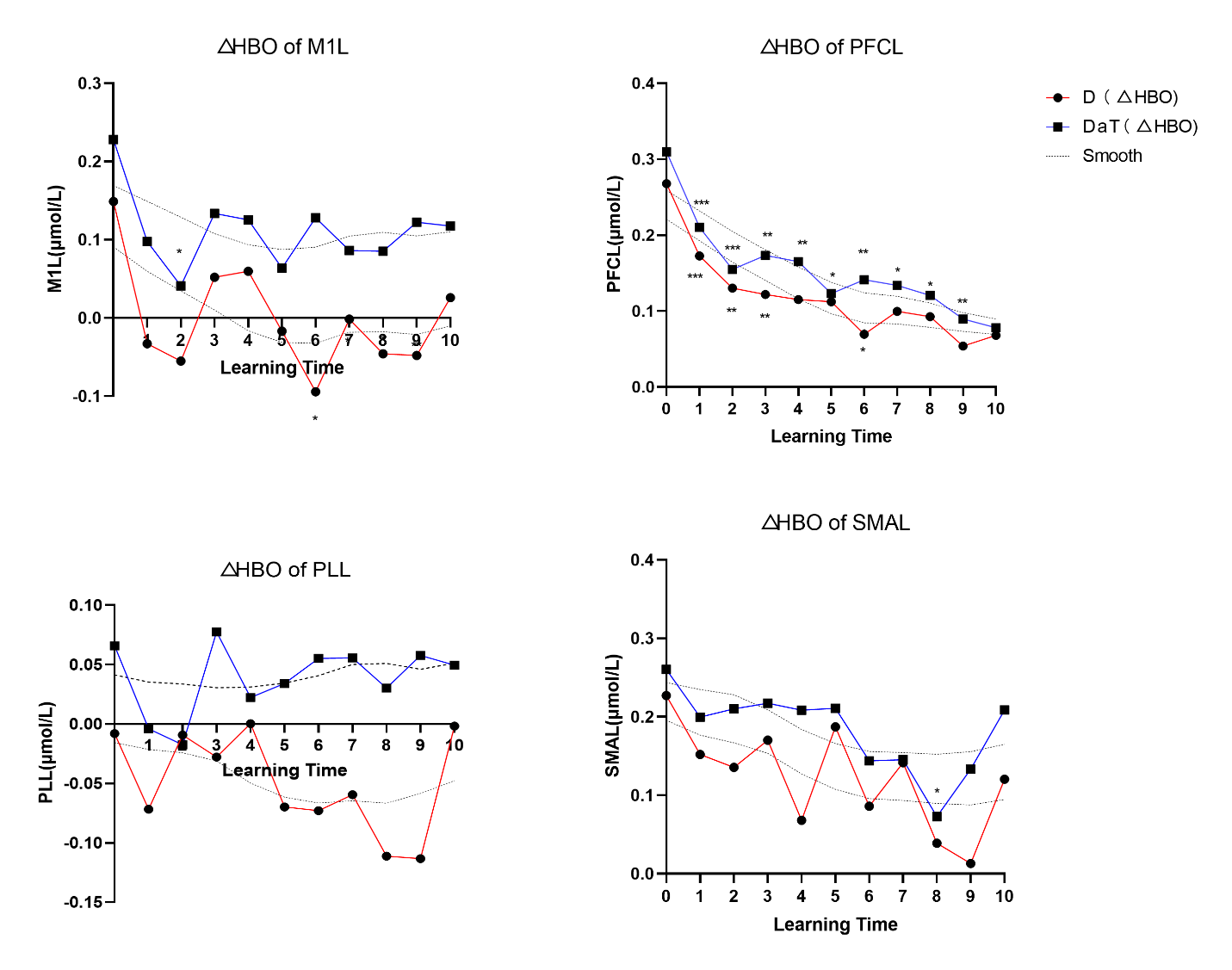


**Supplementary Figure 1: Brain Activation on the Task-performing Side Ipsilateral Brain Regions**

DaT: One Hand Displacing and Turning Task group, illustrated with blue solid lines; D: One Hand Displacing Task group, represented with red solid lines. Vertical axis: The activation of the cerebral cortex was expressed as the change in blood oxygen(ΔHBO,μmol/L); Horizontal axis: Motor learning assessment time points. M1L: Left primary motor cortex; SMAL: Left supplementary motor area; PLL: Left parietal cortex; PFCL: Left dorsolateral prefrontal cortex. * indicates p<0.05, ** indicates p<0.01, *** indicates p<0.001.

**Supplementary Table 1: Brain Activity Differences of the Task-performing Side Ipsilateral Brain Regions Between D and DaT Groups Using GEE （N=31）**

| **Cortical zone** | **TN vs T0** | **D** | | | **DaT** | | |
| --- | --- | --- | --- | --- | --- | --- | --- |
|  |  | **β（s）** | **SE** | **p.value** | **β（s）** | **SE** | **p.value** |
| M1L | T1 vs 0 | -0.1820 | 0.1156 | 0.1154 | -0.1303 | 0.0798 | 0.103 |
|  | T2 vs 0 | -0.2042 | 0.1204 | 0.090 | -0.1870 | 0.0771 | 0.015* |
|  | T3 vs 0 | -0.0970 | 0.0880 | 0.270 | -0.0945 | 0.1098 | 0.390 |
|  | T4 vs 0 | -0.0894 | 0.0933 | 0.338 | -0.1026 | 0.0696 | 0.140 |
|  | T5 vs 0 | -0.1658 | 0.1098 | 0.131 | -0.1642 | 0.0875 | 0.061 |
|  | T6 vs 0 | -0.2432 | 0.1186 | 0.040* | -0.0999 | 0.0980 | 0.308 |
|  | T7 vs 0 | -0.1504 | 0.1401 | 0.283 | -0.1418 | 0.0993 | 0.153 |
|  | T8 vs 0 | -0.1949 | 0.1112 | 0.080 | -0.1427 | 0.0824 | 0.083 |
|  | T9 vs 0 | -0.1969 | 0.1034 | 0.057 | -0.1057 | 0.0690 | 0.126 |
|  | T10 vs 0 | -0.1229 | 0.0955 | 0.198 | -0.1104 | 0.0865 | 0.202 |
| PFCL | T1 vs 0 | -0.1997 | 0.0726 | p＜0.001*** | -0.2316 | 0.0417 | p＜0.001*** |
|  | T2 vs 0 | -0.2139 | 0.0800 | 0.006** | -0.2201 | 0.0550 | p＜0.001*** |
|  | T3 vs 0 | -0.1751 | 0.0895 | 0.008** | -0.1890 | 0.0629 | 0.003** |
|  | T4 vs 0 | -0.1682 | 0.1041 | 0.050 | -0.1758 | 0.0658 | 0.008** |
|  | T5 vs 0 | -0.1984 | 0.0864 | 0.106 | -0.1684 | 0.0722 | 0.020* |
|  | T6 vs 0 | -0.1554 | 0.0838 | 0.022* | -0.1866 | 0.0639 | 0.004** |
|  | T7 vs 0 | -0.1527 | 0.1065 | 0.064 | -0.1443 | 0.0620 | 0.020* |
|  | T8 vs 0 | -0.1461 | 0.0945 | 0.152 | -0.1363 | 0.0652 | 0.037* |
|  | T9 vs 0 | -0.1377 | 0.1043 | 0.122 | -0.1546 | 0.0570 | 0.007** |
|  | T10 vs 0 | -0.0952 | 0.0851 | 0.187 | -0.0991 | 0.0567 | 0.081 |
| PLL | T1 vs 0 | -0.0636 | 0.0972 | 0.513 | -0.0699 | 0.0903 | 0.439 |
|  | T2 vs 0 | -0.0012 | 0.0941 | 0.990 | -0.0834 | 0.0984 | 0.397 |
|  | T3 vs 0 | -0.0198 | 0.0954 | 0.836 | 0.0117 | 0.1121 | 0.917 |
|  | T4 vs 0 | 0.0084 | 0.1055 | 0.937 | -0.0436 | 0.0671 | 0.516 |
|  | T5 vs 0 | -0.0617 | 0.1295 | 0.634 | -0.0317 | 0.0968 | 0.744 |
|  | T6 vs 0 | -0.0649 | 0.1150 | 0.573 | -0.0107 | 0.1368 | 0.938 |
|  | T7 vs 0 | -0.0513 | 0.1221 | 0.674 | -0.0103 | 0.1178 | 0.931 |
|  | T8 vs 0 | -0.1031 | 0.1078 | 0.339 | -0.0356 | 0.0753 | 0.636 |
|  | T9 vs 0 | -0.1052 | 0.1096 | 0.337 | -0.0082 | 0.0937 | 0.930 |
|  | T10 vs 0 | 0.0063 | 0.0930 | 0.946 | -0.0163 | 0.1202 | 0.892 |
| SMAL | T1 vs 0 | -0.0750 | 0.1214 | 0.537 | -0.0607 | 0.0793 | 0.444 |
|  | T2 vs 0 | -0.0917 | 0.1100 | 0.405 | -0.0502 | 0.0719 | 0.485 |
|  | T3 vs 0 | -0.0569 | 0.0870 | 0.513 | -0.0430 | 0.1111 | 0.699 |
|  | T4 vs 0 | -0.1592 | 0.0987 | 0.107 | -0.0517 | 0.0829 | 0.533 |
|  | T5 vs 0 | -0.0400 | 0.0799 | 0.616 | -0.0495 | 0.1020 | 0.628 |
|  | T6 vs 0 | -0.1412 | 0.1005 | 0.160 | -0.1163 | 0.0908 | 0.200 |
|  | T7 vs 0 | -0.0856 | 0.1067 | 0.422 | -0.1150 | 0.0974 | 0.238 |
|  | T8 vs 0 | -0.1882 | 0.0996 | 0.059 | -0.1875 | 0.0724 | 0.010* |
|  | T9 vs 0 | -0.2140 | 0.1189 | 0.072 | -0.1270 | 0.0652 | 0.051 |
|  | T10 vs 0 | -0.1067 | 0.1124 | 0.343 | -0.0515 | 0.0765 | 0.501 |

Based on the GEE model fitting, adjusting for gender, age, and EHI factors. M1L: Left primary motor cortex; SMAL: Left supplementary motor area; PLL: Left parietal cortex; PFCL: Left dorsolateral prefrontal cortex. β represents the relationship coefficient. “-“: Indicates a negative relationship. *: Indicates p<0.05, **: Indicates p<0.01, ***: Indicates p<0.001. SE: Standard error of estimate.

The supplementary materials clearly chart the activation trajectories in ipsilateral cortical regions (M1L, PFCL, SMAL, and PLL) across learning days in both experimental groups. These trajectories are visually represented by solid lines: blue for the DaT group and red for the D group. Notably, we observed a noticeable reduction in activation levels in the M1L and PFCL regions of the right hemisphere, consistent across both groups.

We intentionally focused our main text on contralateral cortical activation patterns, vital for understanding motor learning in the context of potential Transcranial Magnetic Stimulation (TMS) and Transcranial Direct Current Stimulation (tDCS) interventions. This approach aligns with motor control lateralization principles, aiming to enhance rehabilitative strategies. However, recognizing the crucial role of ipsilateral regions in motor learning, we've included supplementary data to offer a comprehensive view, ensuring our research is transparent and complete.

We are ready to further explore this dataset, with a forthcoming publication dedicated to examining bilateral cortical interactions and functional connectivity within the motor learning context. This separate research effort aims to strengthen our knowledge, offering detailed insights into the intricate relationships between cortical regions during motor learning, and informing neuro-rehabilitative interventions. Our dedication to progressing this field is steadfast, and we believe that this phased approach in sharing our findings will maintain scientific integrity, foster targeted rehabilitation methods, and ultimately benefit both the scientific community and patient care.
